# HALT: Relapse prevention to resilience

**DOI:** 10.3389/adar.2025.15477

**Published:** 2026-01-09

**Authors:** Daniel Kretchman

**Affiliations:** Infinity Behavioral Health – Kretchman and Associates, PLLC, North Las Vegas, NV, United States

**Keywords:** addiciton, compulsive behaviors, HALT, recovery, socialcultural factors, resilience, context

## Abstract

The impact of compulsive behaviors (aka addictions), whether substance- or behavioral-based, is substantial. The acronym HALT (Hungry, Angry, Lonely, Tired) has long been used in recovery. This paper provides a discussion of the history and basic concept, with application for relapse prevention and increasing resilience for mental health. It further offers a rationale for expanding this skill set with new and easily applied contextual domains. Using a familiar tool as a building block provides a framework for helping individuals with current or past compulsive behaviors, and for those assisting them. This may decrease relapse incidents which impact the individual and society.

## Introduction

Compulsive behaviors (aka addictions), impact many areas of society and individuals’ lives. The financial, emotional, and social tolls, to name a few, are not likely accurately calculable. The US National Drug Control Budget for 2023 [1] was $43,721,100,000; however, other costs are difficult to estimate, such as the financial cost of reduced productivity, or harm, if someone engages in a compulsive behavior at work. Other effects are difficult or impossible to quantify (e.g., the loss of a child due to overdose, changes in family relationships).

HALT is an acronym for *Hungry*, *Angry*, *Lonely*, *Tired* that is used for relapse prevention in recovery from substance or behavior compulsions (addictions). A search on Google.com conducted on 5/23/2025, for the terms “HALT” and “recovery” returned over 30 million results in 0.26 s.

The concepts embedded in HALT have been part of recovery for many years, notably there is recognition of the impact of physical and emotional states on relapse. Unfortunately, the first use and formulator have been difficult to determine. After searches to identify the origin, using keywords “Halt, hungry, angry, lonely, tired” in web search engines (i.e., DuckDuckGo.com, Google.com) and databases (i.e., EBSCOhost, APA Psycnet, Proquest), this writer reluctantly turned to ChatGPT-40 mini, an Artificial Intelligence (AI) tool on DuckDuckGo.com. The query “What is the origin of HALT for recovery?” on 5/23/2025, resulted in the following response:

The first citation of the HALT acronym in Alcoholics Anonymous (AA) literature is not explicitly documented in the foundational texts like the “Big Book” or “Twelve Steps and Twelve Traditions.” However, the concept of being mindful of emotional and physical states has been a part of recovery discussions since the early days of AA.

The HALT acronym itself became more widely recognized in the 1980s and 1990s, often used in workshops, meetings, and recovery literature to help individuals identify triggers for cravings and potential relapse. While specific citations may not be available, the principles behind HALT have been integrated into various recovery programs and discussions over the years [2].

Many texts, such as Marlatt and Gordon [3] detail components of HALT, without the acronym or specific reference. The earliest sources identified in the above-mentioned searches were in Neurobehavioral Model of Addiction [4], a statement by Bessie Hughs, RN speaking about self-care before the National Commission on Acquired Immune Deficiency Syndrome [5], and Chappel’s work titled *Drug Addiction* [6]. None of these sources provided direction toward the origin. In a text message, Dr. T. Broffman, a retired recovery counselor, classified HALT as “AA folk wisdom” (May 23, 2025).

### Applications

The clinical application of HALT is often focused toward short-term relapse prevention. In other words, when an individual has an urge to use a substance or engage in a behavior, they can use this tool to check in on themselves and possibly identify a factor in their control to help manage the craving.^1^ HALT can also be used for tertiary prevention, to build long-term resilience–potentially helping a person maintain their day-to-day wellbeing and reducing susceptibility to relapse. This is similar to Dialectical Behavior Therapy’s (DBT) *Emotional Regulation* skills (ER) [7] for increasing resilience. The benefit of reducing vulnerability is consistent with research identifying ER difficulties in people with substance use disorders [8] and the evidence of benefit from DBT skills for recovery [9].

While HALT originated in recovery, this writer and others, such as Siegel and Payne Bryson [10], have recognized clinical applications that include, but are not limited to, reducing vulnerability to depression and anxiety, and increasing parenting skills. Although there is limited peer-reviewed research of HALT, the concepts of nutrition, fatigue, emotional regulation, and relationships have been researched and are consistent with other treatment approaches such as DBT [7, 11] and Multimodal Therapy [12].

### Components of HALT

Explanations of the elements of HALT are abundant online, particularly on recovery-related websites (e.g., Dr. D. Streem’s elaboration at https://health.clevelandclinic.org/halt-hungry-angry-lonely-tired [13]). Table 1 provides a synopsis.

**TABLE 1:** Brief summary of HALT components and application.

Component	Addressing urges	Tertiary prevention – long-term resilience
Hungry	Obtain nutrition and hydration	Planned mealtimes, healthy nutrition, and availability of appropriate snacks
Angry Note. Anger may be a secondary emotion, residing beneath hurt or fear [11]	A strong emotion that may lead to relapse as a means of reducing discomfort. As a result, many recovery approaches teach coping skills (e.g., relaxation, re-framing)	Regular practice of stress reduction, reframing, and self-control skills
Lonely	Reach out to identified helpful resources.^2^ Apply learned skills for distress tolerance	Create social supports and activity lists. Develop distress tolerance strategies
Tired	Rest. If not possible at that moment, take time for relaxation	Create routines that provide adequate respite. Identify barriers to good sleep

## Expanding HALT

The theoretical rational for this expansion has roots in Vygotskian theory. This perspective states that looking at the sociocultural context^3^ is necessary for fuller understanding of mental processes [15]. While Vygotsky’s approach dates prior to 1934, we can observe on-going application in many areas of psychology (e.g., the prevalence of diversity training in psychology^4^). Specifically, social supports and influences are often an integral part of recovery and maintenance from compulsive behaviors. Applications include the use of groups for recovery meetings (e.g., 12-step programs, SMART Recovery), and this approach is supported by research, such as (i) Zironi et al.’s [17] work which found that rats who learned to self-administer alcohol in a specific environment and stopped when there were sensory-related changes (i.e., olfactory, visual, and tactile characteristics), resumed alcohol seeking behavior when returned to the original setting; and, (ii) Manca and Lewsey’s [18] findings that identified living situation and socioeconomic status as risk factors in relapse prevention, after first alcohol-related hospitalization. In summary, as Donovan and Chaney state, “simultaneous consideration of person and situational variable explains more variance in human behavior than either does independently, an interactional approach is suggested” [19], p352.

Over time, elements related to sociocultural context have been recognized. This paper collects several of these toward creating an easily accessible tool for maintaining sobriety and good mental health. In learning, applying, and teaching these new components, it may be helpful to keep in mind the concepts of *vulnerability* and *opportunity* as prominent elements within each risk factor.

### Free (availability)

Despite some contradictions [20] there is a prominent body of work that suggests the use of free samples is an effective marketing technique [21], and industries and stores routinely use samples (e.g., Costco, pharmaceutical and cosmetics companies). Old behavioral habits and neural circuitry, combined with “free” samples (e.g., drinks at a party, rewards money for gambling) can present a pathway to past compulsive habits [22]. Relapse prevention and building long-term resilience overlap in addressing this risk factor. Initial work may be done on basic skills, such as identifying situations that place a person at risk for relapse. More sophisticated strategies can include awareness of the hazard of “free” opportunities, identifying individuals that may place them at risk, learning to decline (e.g., through role play), and identifying alternative behaviors.

### Environment/culture

Years ago, someone mentioned to the author that they learned joyful celebrations can lead to relapse. Markose et al. reported that 88% of their study participants relapsed due to “pleasurable events” [23], p28. The concept of joyful events can be generalized to physical environment (e.g., a work gathering with alcohol) and social-cultural events (e.g., holidays, celebrations, funerals). Social occasions have potential for connections associated with past compulsive behaviors, as well as Melemis’s rule-bending opportunities [24]. They can also result in encounters with people who create stress or expectations that can lead to increased vulnerability. The recovery community’s awareness of this risk can be seen in commercial and non-profit website articles that specifically address relapse around holidays (e.g., https://carolinacenterforrecovery.com/addiction-blog/why-is-relapse-so-common-during-the-holidays/ [25]).

### Sick or physical health, including pain

Poor health may present a challenge for maintaining abstinence. Prescribed or unprescribed substances can offer relief, but also present re-exposure that can re-start old behavioral habits and trigger neural circuitry [22]. Furthermore, illness may contribute to other problems like poor self-perception [26], limited access to resources, and isolation. The significance of physical health as a protective measure can be seen in DBT’s *PLEASE* skill [7]. Specific preventative measures include developing strategies to maintain health and identifying resources for times of illness.

### Transitions and stressors

Transitions are changes in different domains such as health, ability, relationships, employment, relocation, legal or government issues, living situation, or loss, to name a few. Negative events are obvious challenges; however, positive changes can also present difficulty, such as the euphoria from a work promotion. Dimensions to explore include Shi and Brown’s transitions that are consistent with our cultural life script (e.g., graduation), and transitions that are divergent from it (e.g., suspension from work) [27]. Transitions are differentiated from stressors in that the former is more objective, and while there is significant overlap not all transitions are stressors and not all stressors involve transitions. Additionally, the objectivity of transition may be easier for individuals to recognize and address. Stressors may be short-term (e.g., an upcoming exam) or long-term (e.g., end of a relationship), and it is important to note that according to some research (e.g., Tate [28]) severe long-term stressors increased the risk of relapse after treatment, while short-term stressors did not. The updated Social Readjustment Rating Scale (SRRS) [29] provides specific areas and quantification, and may allow identification of unrecognized challenges. Prevention includes skills for reframing situations, preparing for known transitions, and reducing reactivity and intensity when responding to unexpected situations. People may also benefit from learning to separate emotions from facts [7, 11] and increasing their ability to identify beliefs that effect their interpretation of the situation.

## Discussion

The goal of this paper is to offer additional skills for recovery. HALT is a commonly used acronym and this expansion provides additional means to (i) reduce the risk of relapse, and (ii) increase long-term resilience for both those in recovery from substance and behavioral compulsions, and people maintaining their mental health. Components are presented as discrete items; however, in real life it is unlikely they will appear in isolation, rather there may be an interaction, such as free alcohol at a celebration.

As the objective is to offer practical techniques, an organized list (e.g., an acronym) allows professionals to provide people with easily recalled strategies in preparation for anticipated situations or unexpected conditions; and, it gives individuals an accessible framework for responding in real-time. As a result, people can learn how a situation may contribute toward an urge, while providing means for responding to cravings and potentially reducing the likelihood of relapse. This article is relatively brief and applications can be explored in groups or individual treatment.

### Future research

Despite wide-spread community use, there is scant research of HALT in the scientific literature, though there is examination of related individual components (for example, see Linehan [11]). The new elements have an accepted theoretical basis, and some supporting research. Assessing each component by itself is possible, albeit somewhat complicated. It may be better to evaluate the entire skill set, or similar to DBT, explore the effectiveness as part of a comprehensive program. Further research avenues include assessment of the impact of the expanded HALT on short-term relapse prevention and long-term resilience building, and application to other mental health conditions.

### Toward a new acronym

Those who work in recovery recognize the need for tools that are easily accessible under duress. As Melemis noted on Gorski’s breakdown of relapse into 11 phases “This level of detail is helpful to clinicians but can sometimes be overwhelming to clients” [24], p325. In its brevity, HALT contains a simplicity that contributes to ease of application. Using this acronym as a foundation may be more beneficial than developing a completely new abbreviation. The acronym HALT FEST (Hungry, Angry, Lonely, Tired, Free, Environment/culture, Sick/health, Transitions/stressors) may be an appropriate option and is used for the handout located online at DrKretchman.com/halthandout.pdf.

## Footnotes

1 The author recognizes this may be a multi-step process, such as: i) recognizing potential for relapse, ii) identifying skill, and iii) successful application of the skill. Elaboration of this process is beyond the scope of this paper.

2 As opposed to reaching out to “relationships where people are only after something from you (like money).” [14].

3 For clarity, the word *context* will refer to any external situational element (e.g., environmental, cultural, societal).

4 At the 2025 American Psychological Association convention over 140 continuing education sessions were available. Of these, excluding age-related categories (e.g., school), approximately 50 topics (35%) included aspects of diversity such as sexual orientation, gender, ability/disability, ethnicity, and/or geographic location [16].
